# AIE-Based Benzothiophene Derivatives as Efficient Latent Fingerprint Detectors

**DOI:** 10.3390/ma19214642

**Published:** 2026-10-31

**Authors:** Patrycja Filipek, Natalia Szczypiorowska, Karol Erfurt, Michał Filapek

**Affiliations:** 1 Institute of Chemistry, Faculty of Science and Technology, University of Silesia, Szkolna 9, 40-007 Katowice, Poland; 2 Department of Chemical Organic Technology and Petrochemistry, Faculty of Chemistry, Silesian University of Technology, Krzywoustego 4, 44-100 Gliwice, Poland

**Keywords:** aggregation-induced emission (AIE), latent fingerprints, donor–π–acceptor, luminescence, benzothiophene

## Abstract

In this work, a series of benzothiophene compounds containing a cyanoacrylate motif was designed and synthesised. A complete physicochemical characterisation was then performed. Optical and electrochemical properties, as well as aggregation-induced emission, were investigated. The analysis was supplemented with theoretical DFT calculations of band gaps, HOMO levels, and LUMO levels. The aggregation-induced emission of the compounds was studied in various mixtures of MeCN, DMF, THF, DMSO, etc. (good solvents) and alcohols, water, and hexane (poor solvents), at varying water contents ranging from 0% to 90%. It was demonstrated that the differences in the physicochemical properties of the obtained compounds due to the length of the alkyl chain in the ester group were not significant for their solutions in good solvents. The opposite situation is observed for the AIE phenomenon (aggregation-enhanced emission), where the length of the alkyl chain in the ester group significantly affected the results. The investigated derivatives were tested for their potential use in latent fingerprint visualisation and material marking.

## 1. Introduction

In 1893, Hans Gross published a manual for investigating judges, the first monograph on forensic science [1]. The primary focus of forensics is the identification of people, animals, or objects based on the traces they leave behind, such as fingerprints or DNA traces. Over the years, latent fingerprint detection has become the basis for identifying a person based on their unique fingerprint pattern. This method is currently very common, using automatic fingerprint identification devices or even smartphones. Initially, only visible fingerprints were detected (for example, being embedded in substances such as blood, grease, or paint). However, over time, there has been a significant increase in the number of fingerprints recovered from crime scenes. This was fueled by the discovery of methods for enhancing invisible fingerprints, which allowed them to be located and visualized [2]. Fingerprint identification is based on the principles of permanence and individuality. This means that a fingerprint does not change over time and is unique to a given person [3]. Fingerprint images are divided into three types: rolled, regular, and latent.

From other side the increase in the number of counterfeit documents, currencies, and even branded clothing is a global problem [4]. Furthermore, the increasing skill level of counterfeiters creates a constant need to develop more effective methods to secure the aforementioned items [5]. Luminescent materials with appropriate optical properties come to the rescue here [6]. The same applies to the detection of latent fingerprints (LFP), which constitute a key piece of evidence in court cases because they are unique to each individual, making them very strong [7]. Current LFP visualisation methods, such as powder dusting or ninhydrin spraying, are often expensive, hazardous for the person performing the identification, or do not produce images with adequate contrast. This last aspect is crucial for the possibility of using LFP as evidence in a case [8]. To address the aforementioned challenges, new structures for potential luminophores are being sought.

In addition to luminescence itself, chemiluminescence—and its variant, electrochemiluminescence—are increasingly used for LFP detection [9,10]. Chemiluminescence is a phenomenon in which light is generated via chemical excitation during a chemical reaction [11]. Briefly, certain substances—such as substrates, intermediates, and fluorophores—are oxidised to form a high-energy oxidised intermediate; this intermediate then decomposes or transfers its energy to nearby fluorophores before returning to the ground state, a process accompanied by the emission of light [12]. Chemiluminescence techniques are widely used to detect blood traces; luminol is a prime example [13].

Donor-acceptor systems demonstrate the most promising properties in this regard. A donor is a molecular fragment capable of donating electrons, while an acceptor is a molecular fragment capable of accepting electrons. In other words, the highest occupied molecular orbital (HOMO) is located on the donor substituent, while the lowest unoccupied molecular orbital (LUMO) is located on the acceptor substituent. Donor-acceptor systems exhibit strong luminescence. This means that upon absorption of a photon, an electron from the donor moves to an unoccupied acceptor orbital, i.e., an intramolecular charge transfer occurs. After the absorbed photon is emitted, the molecules return to their original state, and their structure remains unchanged. These electronic transitions generate an additional (new) band in the absorption spectra, which does not occur in the case of separate components. Typically, this band is located further from the component bands and is very broad and intense [14]. The advantages of donor-acceptor materials include: enormous chemical, thermal, and photochemical stability [15].

An example of structures that may be promising fluorophores for both LFP detection and labelling is benzothiophene-based fluorophores. Benzothiophene-based fluorophores exhibit unique fluorescent properties, including AIE, ICT, mechanofluorochromism, and fluorescence induced by excitation wavelength, pH, or solvent [16].

A significant feature of this work is the aforementioned AIE phenomenon exhibited by benzothiophenes. AIE itself has been extensively studied in recent years due to its extensive use in OLEDs and luminescent probes [17]. This demand for compounds exhibiting AIE is driving a rapid increase in their synthesis. With the intensification of research in the field of AIE, it has been observed that AIE occurs in small molecules. In contrast, AIEE (aggregation-induced enhanced emission) occurs in large molecules, polymers, and a certain percentage of small molecules [18]. The occurrence of the AIE/AIEE phenomenon in a molecule is attributed to a pair of mechanisms: twisted intermolecular charge transfer (TICT) [19], restricted intramolecular rotation (RIR) [20].

This paper presents benzothiophene derivatives that exhibit the AIE/AIEE phenomenon and have potential applications in forensics.

## 2. Materials and Methods

### 2.1. Reagents and Materials

General Methods: All chemicals and reagents were commercially available and were used without further purification. Column chromatography was carried out on Merck Silica Gel 60. Thin-layer chromatography (TLC) was performed on silica gel (Merck TLC Silica Gel 60 F254). More information is presented in the Supplementary Materials. Visualizing the latent fingerprints involved evaporating 100 mg of the specific developer onto silica gel. Subsequently, after depositing a sweat-and-sebum residue onto the surfaces (in this case, cartridge casings), the prepared developer was applied using a brush. Visualization was immediate, and the results were imaged using a TLC plate viewing device.

### 2.2. Synthesis and Characterization

#### 2.2.1. Synthesis of Methyl (2E)-3-(1-benzothiophen-2-yl)-2-cyanoprop-2-enoate


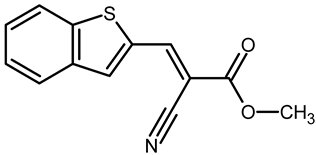


The product was obtained by condensing 100 mg (0.6 mmol) of benzo[b]thiophene-2-carboxaldehyde with 119 mg (1.2 mmol) of methyl cyanoacetate in 70 mL of chloroform in the presence of a few drops of trimethylamine (70 µL) and 10 mg of aluminum oxide. The reactions were carried out at room temperature, under argon, for 18 h. The solvent was then evaporated on a vacuum rotary evaporator. The obtained product was purified by column chromatography on silica gel using methylene chloride as the eluent. The fractions were monitored by TLC. 135.3 mg of yellow product was obtained with a yield of 58%. Rf = 0.79 (CH_2_Cl_2_).

^1^H NMR (500 MHz, CDCl_3_) δ 8.45 (d, *J* = 0.6 Hz, 1H), 8.01 (s, 1H), 7.93–7.88 (m, 2H), 7.52 (ddd, *J* = 8.3, 7.1, 1.3 Hz, 1H), 7.46 (ddd, *J* = 8.1, 7.1, 1.1 Hz, 1H), 3.96 (s, 3H). HRMS (ESI) *m*/*z* calculated for C_13_H_9_NO_2_S [M^+^Na}^+^ 266.0246, found 266.0258.

#### 2.2.2. Synthesis of Tert-butyl (2E)-3-(1-benzothiophen-2-yl)-2-cyanoprop-2-enoate (**bzttBu**)


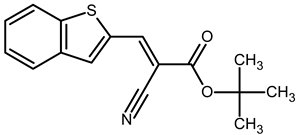


The product was obtained by condensing 200 mg (1.2 mmol) of benzo[b]thiophene-2-carboxaldehyde with 349 mg (2.47 mmol) of tertbutyl cyanoacetate in 100 mL of chloroform in the presence of a few drops of trimethylamine (100 µL) and 10 mg of aluminum oxide. The reactions were carried out at room temperature, under argon, for 18 h. The solvent was then evaporated on a vacuum rotary evaporator. The obtained product was purified by column chromatography on silica gel using methylene chloride as the eluent. The fractions were monitored by TLC. 327 mg of yellow product was obtained with a yield of 93%. Rf = 0.82 (CH_2_Cl_2_).

^1^H NMR (500 MHz, CDCl_3_) δ 8.37 (d, *J* = 0.8 Hz, 1H), 7.99 (s, 1H), 7.95–7.86 (m, 2H), 7.51 (ddd, *J* = 8.4, 7.1, 1.2 Hz, 1H), 7.46 (ddd, *J* = 8.1, 7.1, 1.1 Hz, 1H), 1.62 (s, 9H). ^13^C NMR (126 MHz, CDCl_3_) δ 161.23, 146.64, 142.92, 138.24, 135.75, 134.68, 127.95, 125.53, 125.45, 122.74, 115.68, 103.38, 83.91, 28.00. HRMS (ESI) *m*/*z* calculated for C_16_H_15_NO_2_S [M^+^Na}^+^ 308.0715, found 308.0728.

#### 2.2.3. Synthesis of Octyl (2E)-3-(1-benzothiophen-2-yl)-2-cyanoprop-2-enoate (**bztOc**)


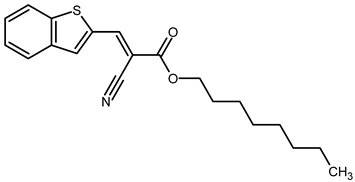


The product was obtained by condensing 200 mg (1.2 mmol) of benzo[b]thiophene-2-carboxaldehyde with 414 mg (2.1 mmol) of octyl cyanoacetate in 100 mL of chloroform in the presence of a few drops of trimethylamine (100 µL) and 10 mg of aluminum oxide. The reactions were carried out at room temperature, under argon, for 18 h. The solvent was then evaporated on a vacuum rotary evaporator. The obtained product was purified by column chromatography on silica gel using methylene chloride as the eluent. The fractions were monitored by TLC. 360 mg of yellow product was obtained with a yield of 58%. Rf=0.87 (CH_2_Cl_2_).

H NMR (500 MHz, CDCl_3_) δ 8.45 (d, *J* = 0.6 Hz, 1H), 8.01 (s, 1H), 7.96–7.88 (m, 2H), 7.52 (ddd, *J* = 8.4, 7.1, 1.2 Hz, 1H), 7.46 (ddd, *J* = 8.1, 7.2, 1.1 Hz, 1H), 4.34 (t, *J* = 6.8 Hz, 2H), 1.92–1.70 (m, 2H), 1.54–1.42 (m, 2H), 1.41–1.21 (m, 8H), 0.92 (t, 1H, *J* = 7.1 Hz, 3H). ^13^C NMR (126 MHz, CDCl_3_) δ 162.50, 147.47, 143.10, 138.19, 135.63, 135.25, 128.16, 125.64, 125.53, 122.76, 115.40, 101.66, 66.90, 31.79, 29.19, 29.17, 28.54, 25.81, 22.66, 14.12. HRMS (ESI) *m*/*z* calculated for C_20_H_23_NO_2_S [M^+^Na]^+^ 364.1341, found 364.1341.

## 3. Results

### 3.1. Synthesis

**Figure 1:**
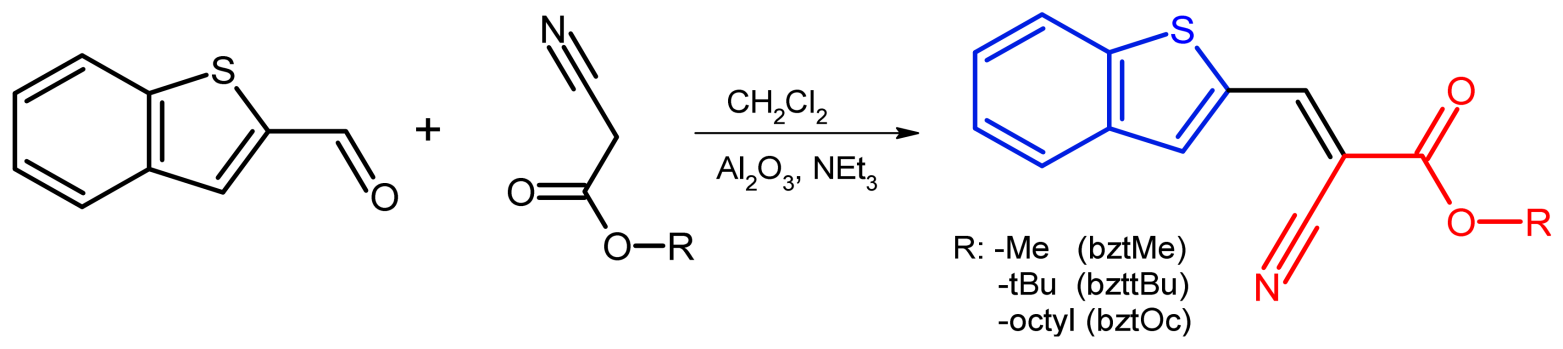
The synthetic route of the obtaining of the investigated compounds. The donor fragment is marked in blue, the acceptor in red, and the linker in black.

Structures of the obtained and evaluated compounds are presented in Figure 1 (below).

During this research, a series of three benzothiophene derivatives was designed and synthesized by condensing benzo[b]thiophene-2-carboxaldehyde with the appropriate cyanoacetate (with a few drops of triethylamine as the reaction initiator) and calcined alumina (to bind the water, which is a byproduct in this reaction). All derivatives belong to the donor-pi-acceptor group of compounds. In this case, the benzothiophene moiety (marked in blue in Figure 1) served as the donor, a cyanoester (red part of the molecule) was incorporated into the molecular structure as the acceptor, and vinyl was used as the linker. This resulted in a system that allows for free charge flow within the molecule. On the other hand, the ester substituents should not affect the molecule’s energy parameters but should significantly influence intermolecular interactions. After isolating the pure derivatives, ^1^H NMR analysis was performed. On the one hand, this method was used to confirm the structure and purity of the compounds, but on the other hand, to verify whether the assumptions mentioned above are correct. As is commonly known, hydrogen shifts in the NMR spectrum result directly from their environment, i.e., electron density. The hydrogen shifts in the aromatic and vinyl regions are as follows: δ 8.45, 8.01, 7.93–7.88, 7.52, 7.46 (bztMe); 8.37, 7.99, 7.95–7.86, 7.51, 7.46 (bzttBu), and 8.45, 8.01, 7.96–7.88, 7.52, 7.46 (bztOc). Therefore, the only difference worth mentioning is observed for the vinyl signal in the bzttBu molecule (a 0.08 ppm shift relative to the other derivatives). The remaining signals remain practically identical, confirming that the ester substituent does not actually affect the electronic properties of the molecule.

### 3.2. DFT Calculations

Theoretical studies provide an excellent complement to the experimental studies. Quantum chemistry calculations were performed for the compounds presented in this work using the B3LYP [21,22,23,24] functional and the 6-311+G** [25,26,27] basis set. Structure optimisation was performed in vacuum. Based on these geometries, the frontier orbitals of molecules in dichloromethane were calculated (Table 1).

The first step was to analyse the frontier orbitals. As mentioned before, all of the compounds are DA systems in which the donor moiety is benzothiophene and the acceptor moiety is 2-cyanoacrylate ester. The HOMO orbital is located primarily on the donor moiety of the molecule and to a lesser extent on the acceptor moiety. The LUMO orbital is located primarily on the ester (acceptor) moiety and to a lesser extent on the donor moiety, i.e., the benzothiophene (Table 2). The effect of the alkyl chain length on the 2-cyanoacrylate ester unit is not significant. This is evident by comparing the ionisation potential (IP) and electron affinity (EA) values. All molecules exhibit similar IP values in the range of 6.43–6.47 eV. The lowest value is for the bzttBu derivative with a tert-butyl chain, and the highest for bztMe with a short methyl chain (Table 1). The same applies to the EA values, which range from 3.22 to 3.28 eV. The calculated Eg(HOMO-LUMO) gaps confirm the small differences between the HOMO and LUMO levels associated with different alkyl bond lengths in the 2-cyanoacrylate ester unit. Importantly, the gaps identified in theoretical calculations are consistent with those observed in optical and electrochemical measurements.

### 3.3. Electrochemical Properties

The molecules were designed and synthesized with the architecture ensuring that their physicochemical parameters (namely, HOMO and LUMO levels and band gap) are approximately the same across this series. However, the introduction of different substituents in the ester group should dramatically affect the type and strength of intermolecular interactions. Considering the results of DFT calculations, this approach was successful, and the resulting derivatives exhibit very similar values of the frontier orbitals calculated for single, isolated molecules. To confirm these results, a series of electrochemical measurements was performed, primarily using cyclic voltammetry in dilute solutions. This method allows, by extrapolating the linear portion of the reduction peak, the determination of the EA value for a single molecule. Similarly, in the case of oxidation, the determination of the IP value. These values are convertible to absolute values (i.e., to vacuum energy) using ferrocene as an internal standard, thanks to the knowledge of its IP (in this work, the value of the fc/fc+ pair was assumed to be as high as −5.1 eV [28]).

**Figure 2:**
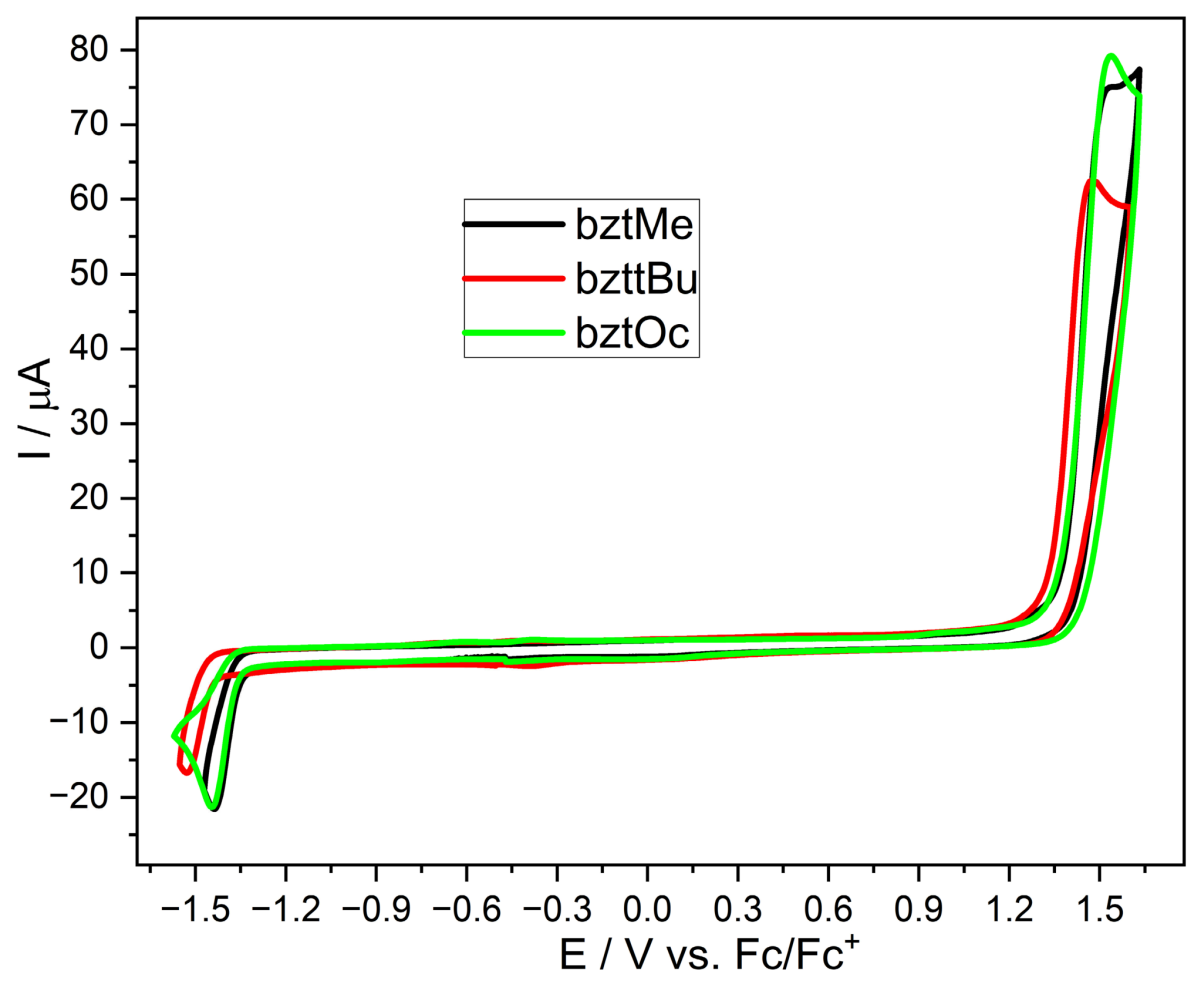
Cyclic voltammograms of the investigated compounds with sweep rate ν = 100 mV/s, 0.1 M Bu_4_NPF_6_ in CH_2_Cl_2_.

As can be seen in Figure 2, upon oxidation, all derivatives behave almost identically, with Eox in each case between 1.29 V (bzttBu) and 1.34 V (bztMe). The effect of substituents is therefore very small, though noticeable, and is due to an increase in the electron-donating power of the substituents in the Me<octyl<tBu series. They stabilize the resulting cation radical, thus lowering the external potential necessary for oxidation. Similar relationships were observed in the reduction study, where a similar effect of substituent changes was also highlighted. However, it should be remembered that in this process, increasing the substituent’s donating power lowers the potential necessary for reduction (the electron density of the molecular fragment accepting the electron increases). However, as can be seen from the observed numerical values (E_red_ from −1.35 V to −1.43 V), this effect is stronger than for E_ox_, meaning that the substituents are closer to the acceptor than to the donor portion. This clearly aligns with the predictions that the LUMO orbital is mostly located on the cyano-ester portion of the molecule. As mentioned, the differences are not large, reaching a maximum of 80 mV, which translates into energy gaps in this series ranging from 2.69 to 2.72 eV. These values are 0.5 eV lower than those determined theoretically (3.19 to 3.21 eV). It is worth noting that the IP calc and experimental values are very similar (see Table 3), but the difference is almost exclusively due to the discrepancy in the EA energy, a common observation. Therefore, summing up this stage of research, it has been demonstrated that the effect of substituents on the energy parameters is not significant, as expected.

### 3.4. Optical Properties

**Figure 3:**
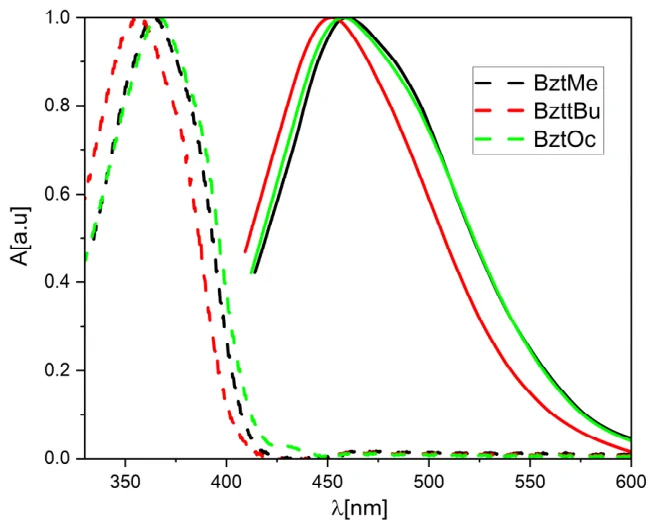
Normalized electronic absorption (dashed lines) and PL spectra (solid lines) of the investigated compound in CH_2_Cl_2_ solution (λ_ex_ = λ_max(abs)_, c = 1 × 10^−5^ M).

DFT calculations confirmed that the HOMO orbital lies primarily on the pi-excess moiety, while the LUMO orbital lies on the acceptor moiety. The linker used to connect these two moieties (i.e., a conjugated system of double and single bonds) ensures efficient charge transfer during excitation (IMCT). All compounds are yellow, meaning their complementary color is blue in the 400–450 nm range. Indeed, when dissolved in acetonitrile, these compounds absorb light at wavelengths at the high-energy end of the spectrum, i.e., 356, 363, and 364 nm for bzttBu, bztMe, and bztOc, respectively (see Figure 3). There are, therefore, subtle, albeit small, differences in properties, with the methyl and octyl derivatives being almost identical, while the tert-butyl-containing molecule differs slightly—both the λ_max_ (abs) and the onset peak are the lowest in energy. This is obviously due to its highest electrodonor activity, as this group contains as many as nine protons bound to the alpha carbons, which gives it exceptionally strong stabilization through hyperconjugation. It is worth remembering that this substituent is located directly next to the acceptor group of the molecule, so it lowers the LUMO level and, consequently, increases the energy gap (Eg).

During the purification process (when exposing TLC plates to a UV lamp), intense emission from the obtained compounds was observed, significantly higher (even to the naked eye) for the solid than for the solution. Therefore, a series of fluorimetric measurements was performed. Considering the possibility of AIE for these compounds, acetonitrile was selected as the solvent for preliminary fluorimeter tests, while the wavelength at which the absorption maximum is found was chosen as the excitation wavelength. It is worth noting that this range is ideal for potential applications in the discovery of latent traces or document marking, as commercially available, portable, and inexpensive UV LED flashlights emit light at wavelengths of 360–366 nm. As can be seen in Figure 3, all compounds emit light in the visible range after irradiation, specifically at 452, 459, and 460 nm (for bzttBu, bztMe, and bztOc, respectively). Again, significant similarities can be seen between the derivatives, especially between the methyl and octyl esters. The total Stokes shift for this group is approximately 100 nm. The emission quantum yields under these conditions (i.e., for diluted solutions) were quite low, i.e., >1%, which, compared to the clearly higher values for the solid, allowed us to assume that this series of derivatives would show a large, positive AIE phenomenon.

### 3.5. AIE Studies

The AIE study began by selecting the best good/bad solvent system. Various systems of both good solvents (MeCN, DMF, THF, DMSO, etc.) and bad solvents (alcohols, water, hexane) were tested. Ultimately, the best results were obtained for the water/MeCN pair, which is a very common choice [29,30,31]. Detailed measurements began with 3D scan measurements to determine the locations of all (quantum-efficient) λ_ex_/λ_em_ pairs. In the case of the described group of compounds, we can distinguish at least three such pairs (including two clearly separated ones).

**Figure 4:**
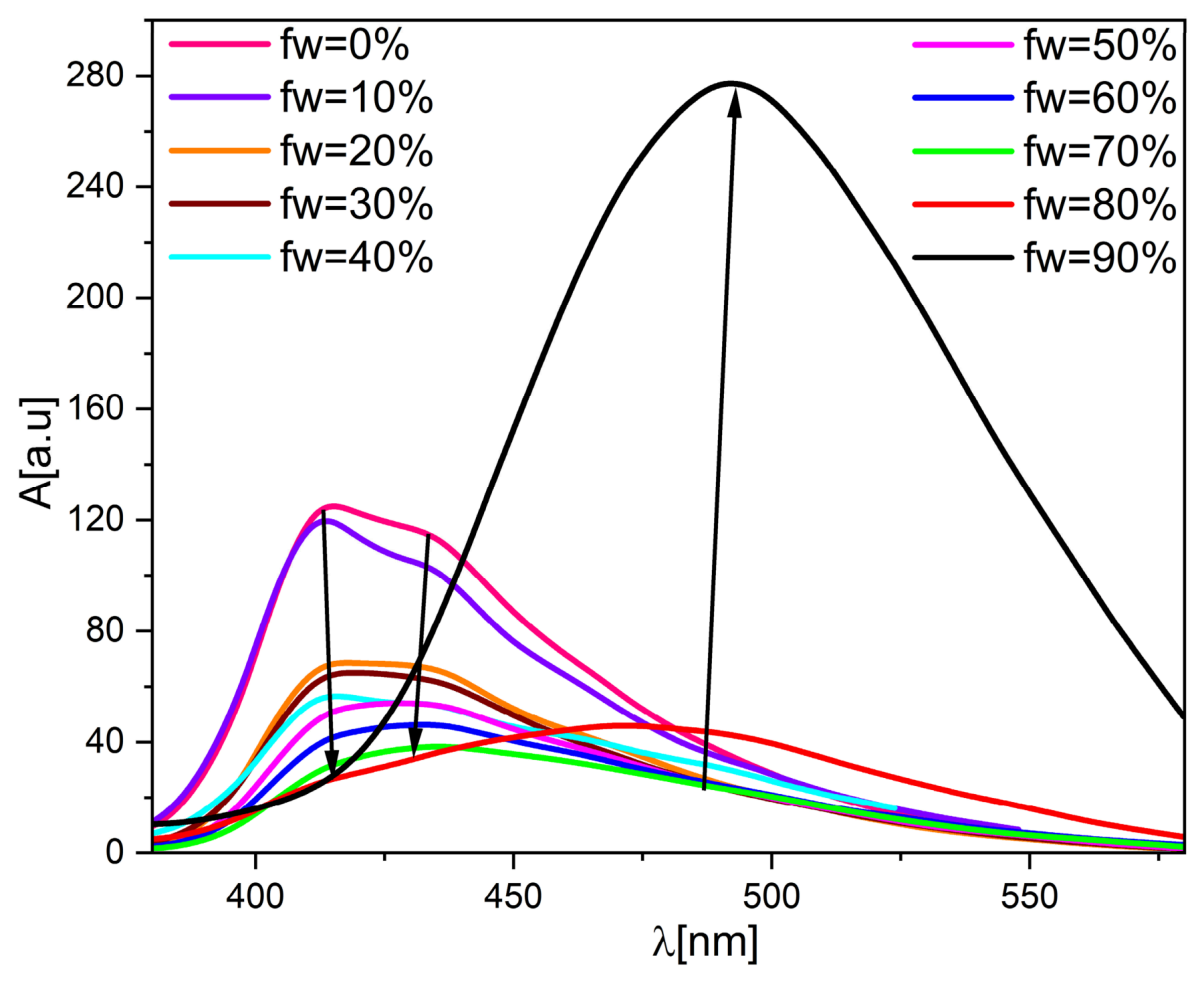
PL spectra of the **bztMe** compound in MeCN/water solution (λ_ex_ = 299 nm; c = 1 × 10^−5^ M).

In the case of the methyl derivative bztMe, the most efficient emission is for excitation at 299 and 360 nm. For solutions in pure acetonitrile, excitation at λ = 299 nm causes emission of the light with two distinct maxima, i.e., at 415 and 433 nm and with quantum yields (QY) = 28.8%. Increasing the water content causes a gradual lowering of the emission quantum yield in the first phase (see Figure 4), with a minimum value of 11.8% for 70% water content. After exceeding it, the emission efficiency increases rapidly, but only for light with emission λ_max_ equal to 492 nm and quantum yield equal up to 85.6%.

When excited at 360 nm (see Figure S1), the molecule exhibited similar behavior. The initial emission band, with a maximum at 458 nm and an efficiency of 7.4%, loses intensity and undergoes redshifting as the fw increases, reaching a minimum quantum efficiency of 6% for a 70% water content. For this solvent mixture composition, the emission has two distinct peaks—at 473 and 492 nm. Further increasing the water content to 80% causes an increase in intensity (to 9.4%) while maintaining the two-maximal characteristic described above. A significant qualitative and quantitative change occurs at fw = 90%, where a single peak clearly dominates the band, with a maximum at 494 nm and an efficiency of 57%.

**Figure 5:**
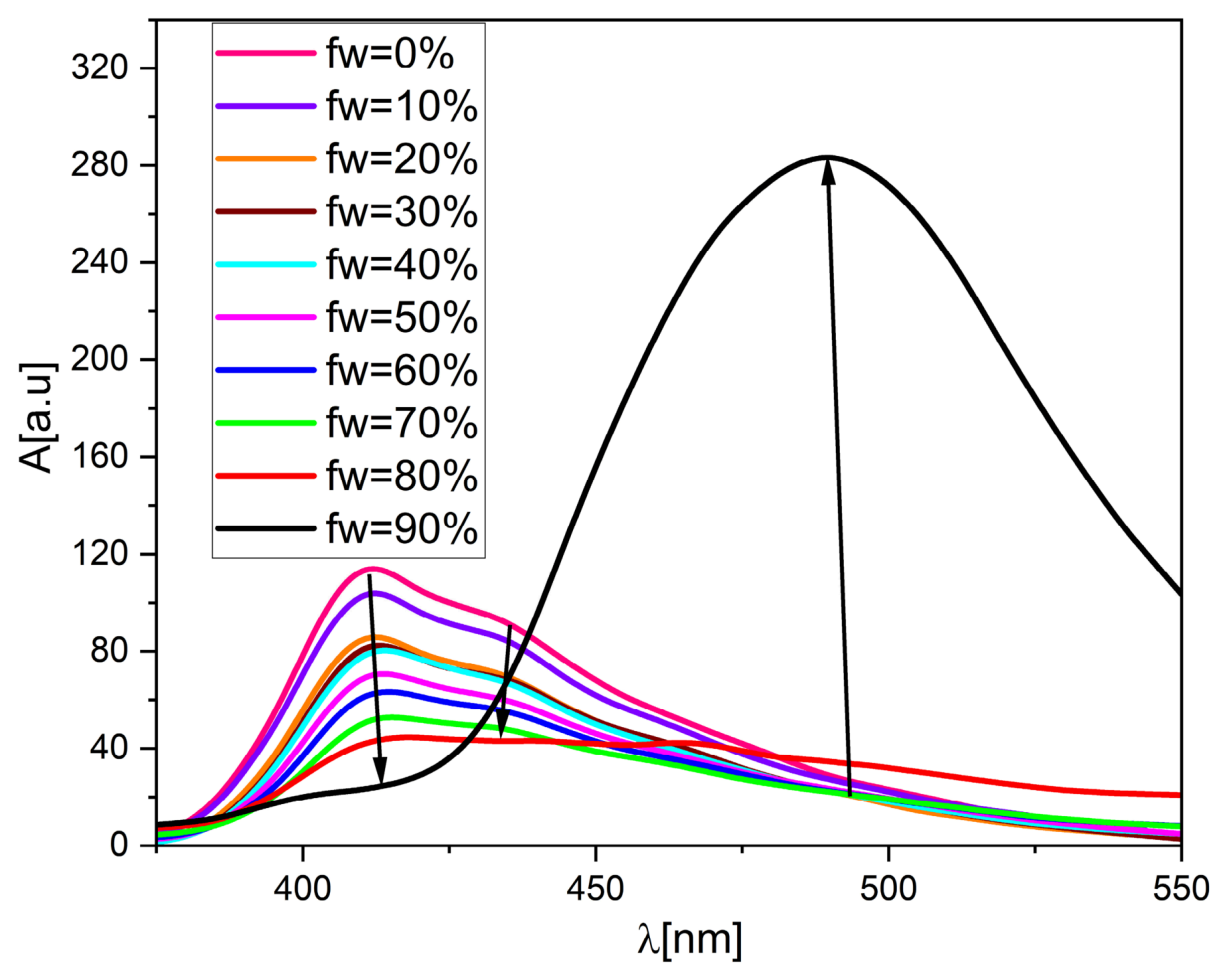
PL spectra of the **bzttBu** compound in MeCN/water solution (λ_ex_ = 284 nm; c = 1 × 10^−5^ M).

A solution of the tert-butyl derivative in pure acetonitrile, when excitated at 284 nm—Figure 5, also emits light in the visible range with two distinct maxima at 412 nm and 434 nm, with an overall quantum yield of 25.8% (the transition with the maximum at around 460 nm is also faintly visible). As with the methyl derivative, increasing water content initially causes a decrease in the emission quantum yield, to 14.8% at fw = 70%. After exceeding this value (for fw = 80%), the yield increases to 17.8%, and the band structure takes on an intermediate appearance, with four maxima (at 412, 434, 464, and 489 nm). For 90% water content, the yield is highest, reaching 78%, with the transitions occurring for the molecule in the pure solvent completely disappearing.

We also analyzed the changes in the emission spectrum of this derivative as the solvent ratio was changed under 360 nm excitation—Figure S2. In pure acetonitrile, the spectrum is simple, with a single maximum at 452 nm and a yield of 7.2%. Again, increasing the water content causes a slight decrease in yield to 6.4% and a change in the spectral characteristics, with two distinct peaks appearing at 461 and 489 nm. Increasing the fw to 90% transforms the spectrum back to a simple form with a single dominant transition (at 489 nm), but with a significantly higher yield of 68%.

**Figure 6:**
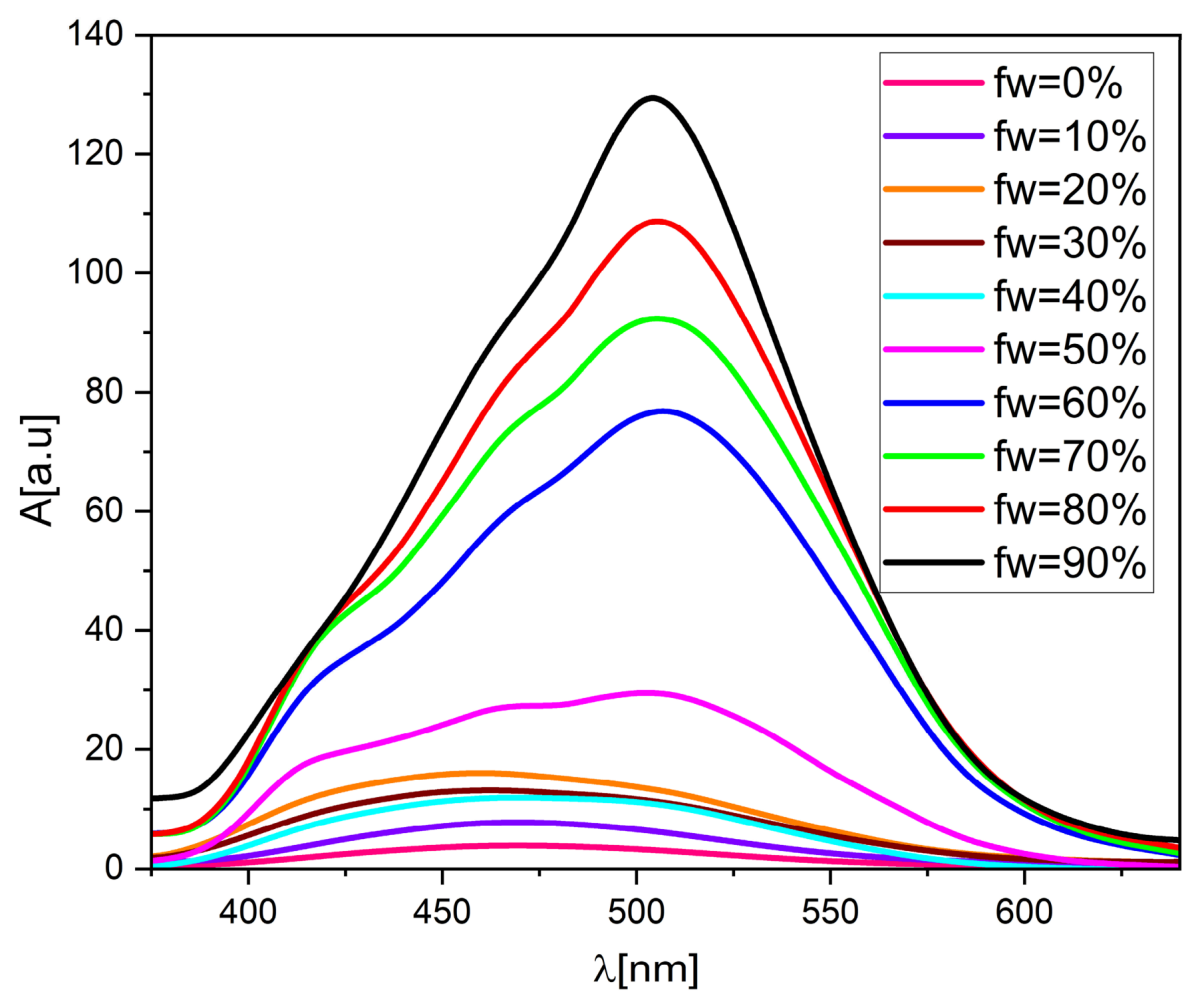
PL spectra of the **bztOc** compound in MeCN/water solution (λ_ex_ = 284 nm; c = 1 × 10^−5^ M).

The last of the tested derivatives differs slightly from those previously discussed. First of all, in solution in pure acetonitrile, it is almost non-emitting (1.6% for the maximum peak at 469 nm)—Figure 6. Increasing the water content from 10% to 20% causes a slight increase in efficiency, to 3.2% and 7.4%, respectively. However, it decreases for a solution with water content = 30% (to QY = 6.2%), and even to 4.8% for fw = 40%. For a solution with 50% water content, the emission quantum yield increases to 12.8%, and the spectrum becomes composed of three transitions with maxima at 415, 460, and 504 nm. Interestingly, for fw = 60%, the quantum yield jumps to 30.8%, and further increasing the water content from this point onwards results in a relatively steady increase in efficiency to 37.9%; 42.0% and 47.4% (for solutions with fw = 70, 80 and 90%, respectively). In turn, for emission induced by 360 nm light in pure MeCN, the ortyl derivative emits light with a maximum at 457 nm and an efficiency of 7.6%—Figure S3. Increasing the water content in this case causes a very small, gradual decrease in quantum yields to 6.4% (fw = 40%). Beyond this level, there is a systematic increase in QY up to 48.4% for the system with 90% water content.

To summarize this part of the study, the significant positive impact of aggregation on emission quantum yield is worth noting. As is the case with almost all molecules reported so far, the group of compounds we investigated also features a system that can rotate about a single bond. This leads to energy loss following excitation through non-radiative transitions [30]. After aggregation, intermolecular interactions among the AIE molecules restrict rotor rotation, causing the molecules to decay via radiative channels. Moreover, in every case, for the investigated compound, we observed a shift in the emitted light toward lower-energy wavelengths. This is, of course, a result of the formation of J-aggregates, a phenomenon well-documented in the literature [31].

### 3.6. Preliminary Forensic Research

**Figure 7:**
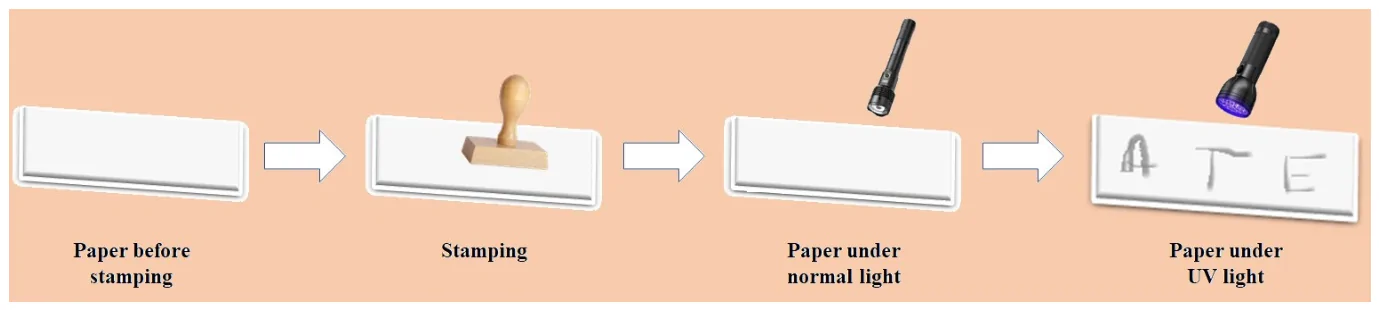
Graphical representation of document marking.

Organic heterocyclic luminogens, whose fluorescence is based on aggregation-enhanced emission, are finding increasingly widespread applications in forensics, including as competitive agents for latent fingerprint detection, drug detection, and document marking [32]. Until now, the AIE structures developed by our group have been applied to latent fingerprint detection [33,34], but those presented in this paper will also be applied to document marking—Figure 7. With increasing globalisation, the problem of document counterfeiting has become increasingly accurate; finding appropriate dyes is necessary [7].

**Figure 8:**
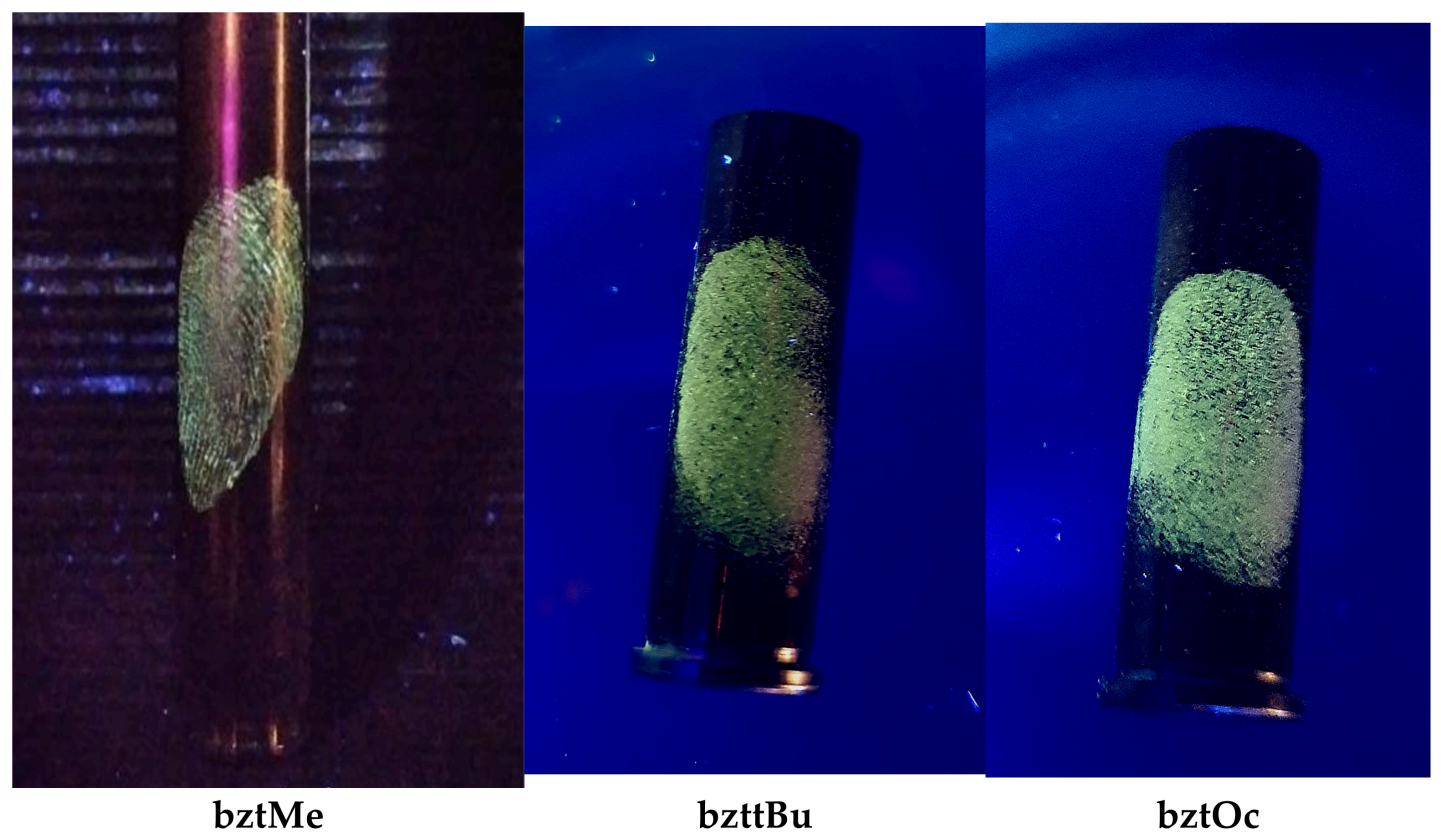
Photo of a latent fingerprint visualization under UV light (365 nm) using **bztMe**, **bzttBu**, **bztOc**.

In this paragraph we will focus on three surfaces: paper, leaf, and banknote (see the Table 4) and LFP (see Figure 8). The application method was the same as in the diagram. No emission enhancement was observed for the paper surface. Only a contrast between the surface and the paper application/marking site was observed. The highest contrast was obtained for t-Bu. In the case of a natural surface—a leaf—emission enhancement was observed after application of the substance, and the best effect was also obtained for the substance containing a tert-butyl substituent. In the case of the final substrate, a banknote, emission enhancement was also observed after application to the surface. The best contrast was obtained for the octyl substituent. For LFP, the best result was obtained for the bztMe derivative. As shown in Figure 8, a complete LFP image was obtained for bztMe, providing a map that confirms surface contact; furthermore, visualisation at the third level revealed complex fingerprint patterns such as whorls or central loops, whereas the second level showed only random patterns lacking key structural features. The first level merely confirms finger-to-surface contact. Satisfactory results were obtained for the other two derivatives, bztt-Bu and bztOc, though they were not as good as those for the first derivative. Both derivatives confirm contact with the substrate—corresponding to the previously described first level—but show no LFP details.

## 4. Conclusions

As part of this work, three donor-acceptor derivatives were obtained, in which the benzothiophene motif acted as the donor and the methyl, octyl, and tert-butyl esters substituents acted as acceptors. To verify the effect of the substituent on the benzothiophene core, optical and electrochemical measurements were performed. These measurements were combined with theoretical DFT calculations and clearly demonstrated a minor effect of the substituent on the benzothiophene core. Although the introduced substituents did not affect the energy parameters of the molecule, they significantly influenced the AIE phenomenon observed for this group of compounds. For the short-chain derivatives (bztMe and bzttBu), it was shown that an initial increase in fw causes a decrease in quantum yield. However, the quantum yield increases rapidly for fw = 90%, but at the same time, the emitted light is shifted by approximately 80 nm toward lower-energy light. Interestingly, in the case of the derivative with a long, unbranched octyl chain, the derivative behaves in a much more similar way to the “classical” AIE phenomenon. i.e., the low initial emission value remains almost unchanged until it exceeds a limit value, after which the molecules aggregate into larger, more emissive systems. Practical tests revealed that the best contrast—in the context of marking on various surfaces—was obtained for bzttBu (natural surfaces such as a leaf) and bztOc (for a banknote). In the case of latent fingerprints, the derivative containing the bztMe motif performed best.

## Figures and Tables

**Table 1:** IP, EA, and energy gap values for **1**–**3**.

Code	IP [eV]	EA [eV]	Eg [eV]
bztMe	−6.47	−3.28	3.19
bzttBu	−6.43	−3.22	3.21
bztOc	−6.46	−3.26	3.20

**Table 2:** The HOMO and LUMO levels.

Compound	HOMO	LUMO
bztMe	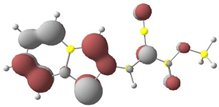	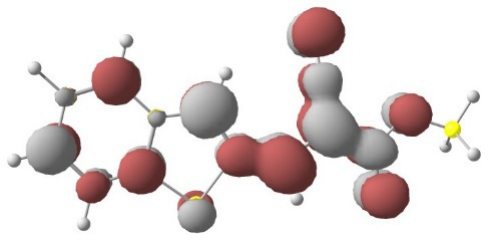
bzttBu	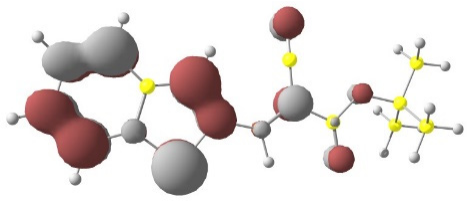	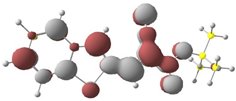
bztOc	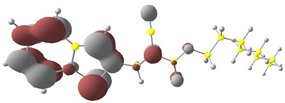	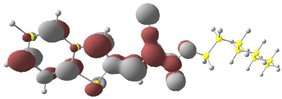

**Table 3:** IP, EA, and energy gap values for **1**–**3**.

Code	E_ox_	E_red_	IP ^a^	EA ^a^	Eg (cv) ^a^	λ_onset_ [nm]	Eg (Opt) ^b^	IP ^c^ [eV]	EA ^c^ [eV]	Eg ^c^ [eV]
bztMe	1.34	−1.35	−6.44	−3.75	2.69	410	3.02	−6.47	−3.28	3.19
bzttBu	1.29	−1.43	−6.39	−3.67	2.72	414	2.99	−6.43	−3.22	3.21
bztOc	1.32	−1.36	−6.42	−3.74	2.68	412	3.01	−6.46	−3.26	3.20

^a^ calculated from CV measurements (IP = −5.1 − E_ox_; Ea = −5.1 − E_red_; Eg_(CV)_ = E_ox (onset)_ − E_red (onset))_; ^b^ obtained from UV-Vis using Eg = 1240/λ_onset_ formula; ^c^ obtained from DFT.

**Table 4:** Fluorescent images of labeled objects (upper in day light, lower in UV (365 nm).

Compound/Material	Paper	Leaf	Banknote
bztMe	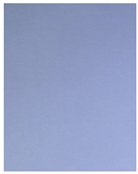	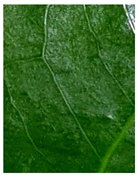	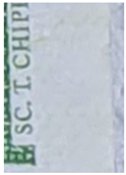
	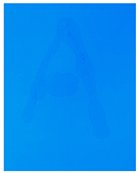	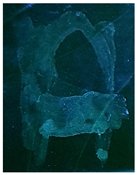	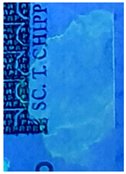
bztOc	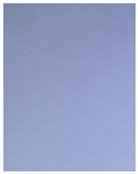	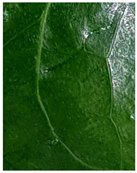	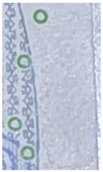
	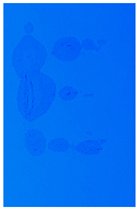	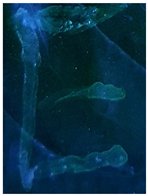	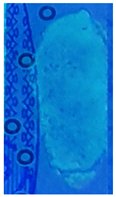
bzttBu	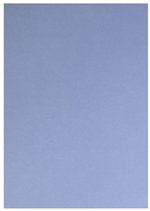	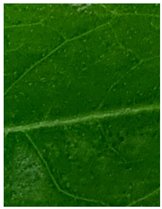	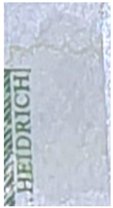
	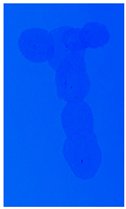	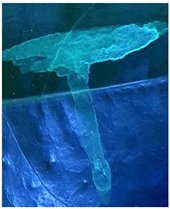	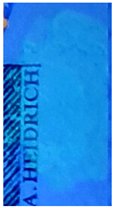

## Data Availability

The original contributions presented in this study are included in the article/supplementary material. Further inquiries can be directed to the corresponding authors.

## Supplementary Materials

The following supporting information can be downloaded at https://www.mdpi.com/article/10.3390/ma19214642/s1, **Spectrum S1.** ^1^H NMR spectrum of (bztMe) (in CDCl_3_): **Spectrum S2.** ^13^C NMR spectrum of (bztMe) (in CDCl_3_). **Spectrum S3.** ^1^H NMR spectrum of (bztMe) (in CDCl_3_). **Spectrum S4.** ^13^C NMR spectrum of (bztMe) (in CDCl_3_). **Spectrum S5.** ^1^H NMR spectrum of (bztMe) (in CDCl_3_). **Spectrum S6.** ^1^H NMR spectrum of (bztMe) (in CDCl_3_). Figure S1. PL spectra of the **bztMe** compound in MeCN/water solution (λ_ex_ = 360 nm; c = 1 × 10^−5^ M). Figure S2. PL spectra of the **bzttBu** compound in MeCN/water solution (λ_ex_ = 360 nm; c = 1 × 10^−5^ M). Figure S3. PL spectra of the **bztOc** compound in MeCN/water solution (λ_ex_ = 360 nm; c = 1 × 10^−5^ M) [35].
