# Supplementary material for: AIE-Based Benzothiophene Derivatives as Efficient Latent Fingerprint Detectors
Source: Materials (Basel). 2026 Oct 31;19(21):4642. doi: 10.3390/ma19214642 (PMC13765406; doi:10.3390/ma19214642)
Supplement: Supplementary file 1 [file materials-19-04642-s001.zip › materials-4588242-supplementary.pdf]

Supporting Information

AIE-based benzothiophene derivatives as efficient latent fingerprint detectors

Patrycja Filipek, Natalia Szczypiorowska, Karol Erfurt, Michał Filapek

Table of Contents

1. NMR spectra 2
1.1. Methyl (2E)-3-(1-benzothiophen-2-yl)-2-cyanoprop-2-enoate (**bztMe**) 2
1.2. Tert-butyl (2E)-3-(1-benzothiophen-2-yl)-2-cyanoprop-2-enoate (**bzttBu**) . 3
1.3. Octyl (2E)-3-(1-benzothiophen-2-yl)-2-cyanoprop-2-enoate (**bztOc**) 4
2. Materials and Methods 5
2.1. Reagents and materials 5
3. Optical properties 6

1. NMR spectra

1.1. Methyl (2E)-3-(1-benzothiophen-2-yl)-2-cyanoprop-2-enoate (**bztMe**)

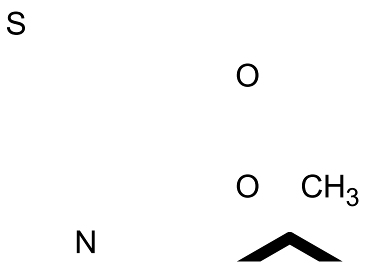

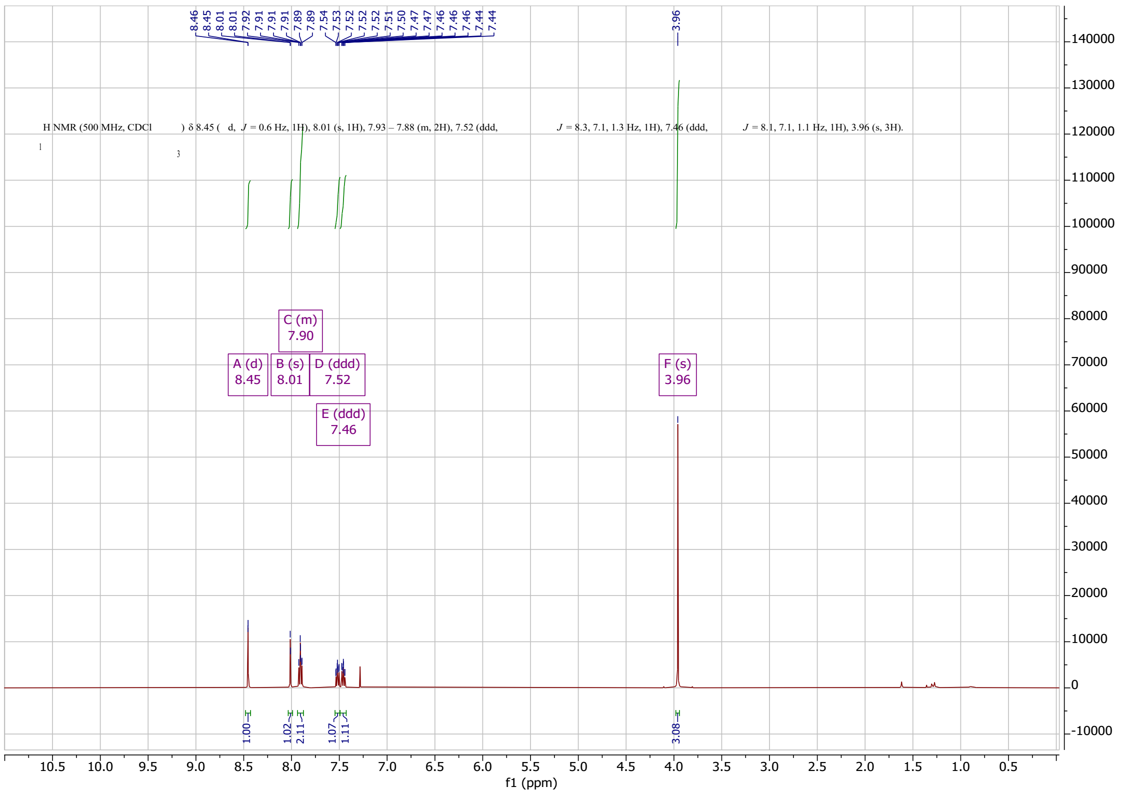

Spectrum S 1. ^{1}H NMR spectrum of (bztMe) (in $CDCl_3$).

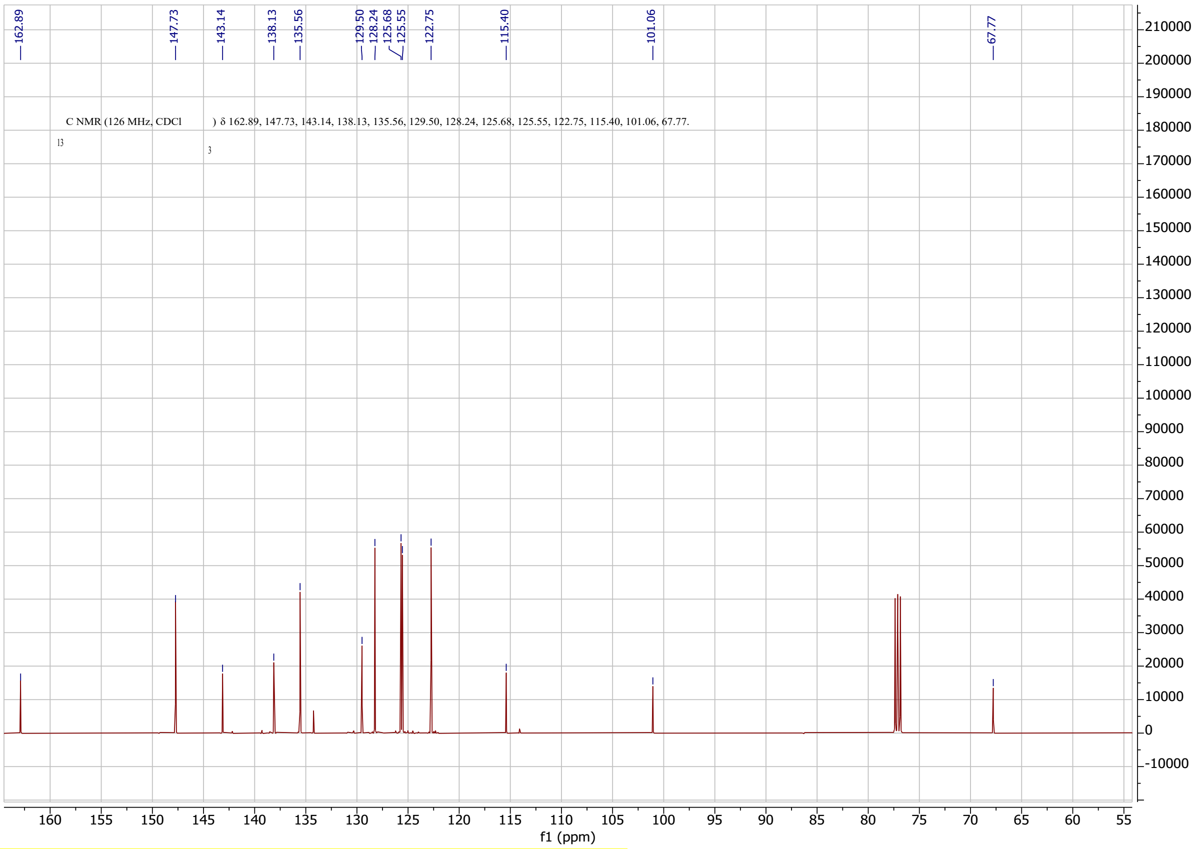

Spectrum S 2. ^{13}C NMR spectrum of (bztMe) (in $CDCl_3$).

1.2. Tert-butyl (2E)-3-(1-benzothiophen-2-yl)-2-cyanoprop-2-enoate (**bztt-Bu**)

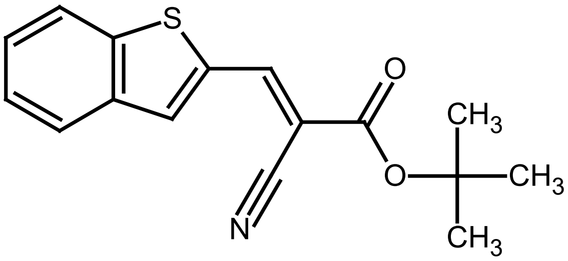

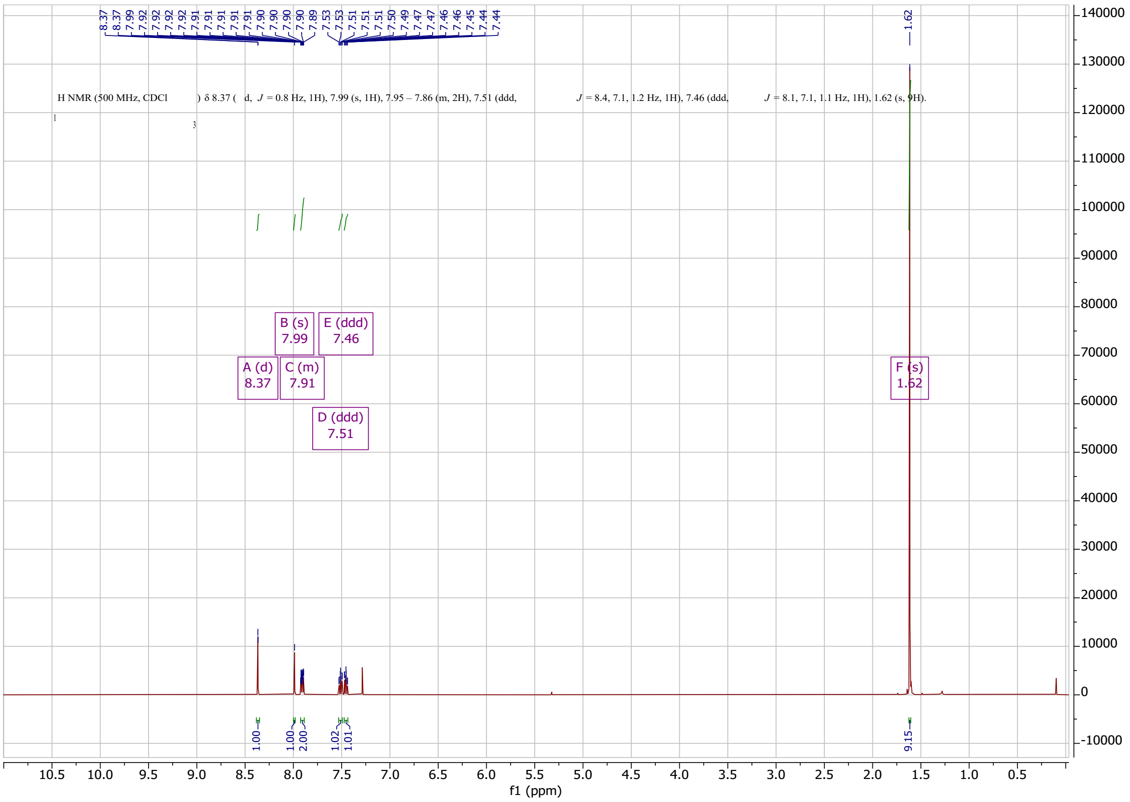

Spectrum S 3. ^{1}H NMR spectrum of (bztMe) (in $CDCl_3$).

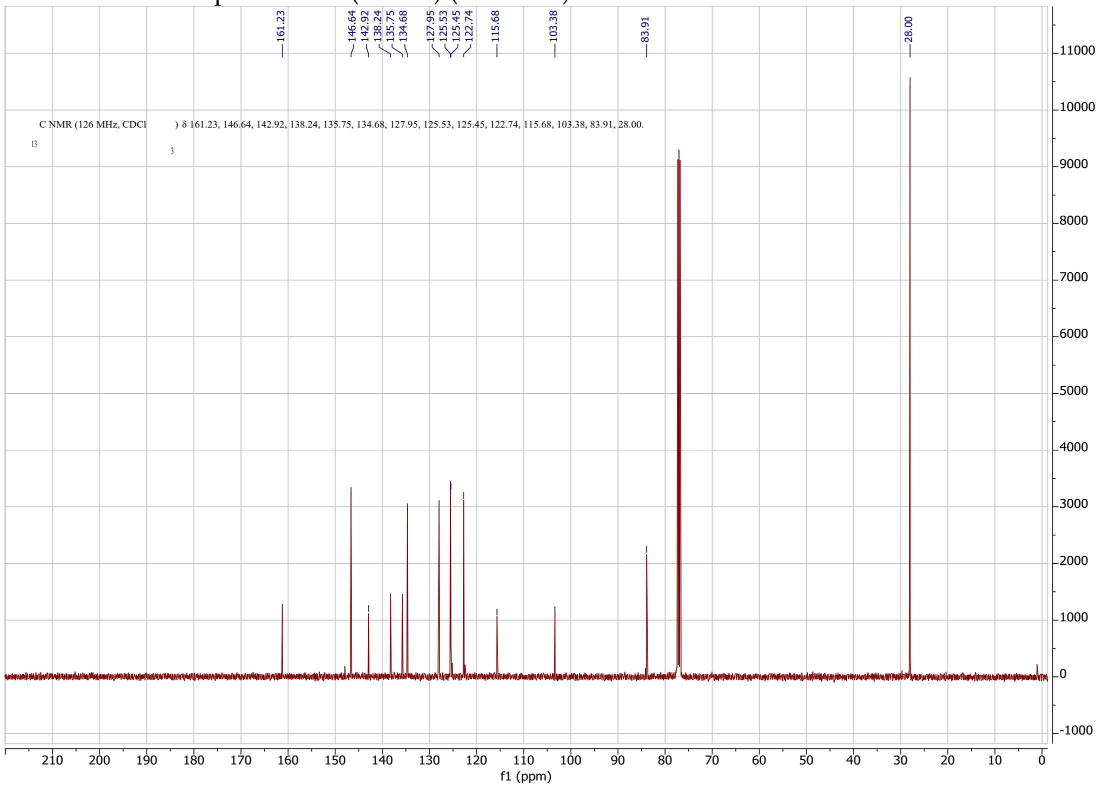

Spectrum S 4. ^{13}C NMR spectrum of (bztMe) (in $CDCl_3$).

1.3. Octyl (2E)-3-(1-benzothiophen-2-yl)-2-cyanoprop-2-enoate (**bztOc**)

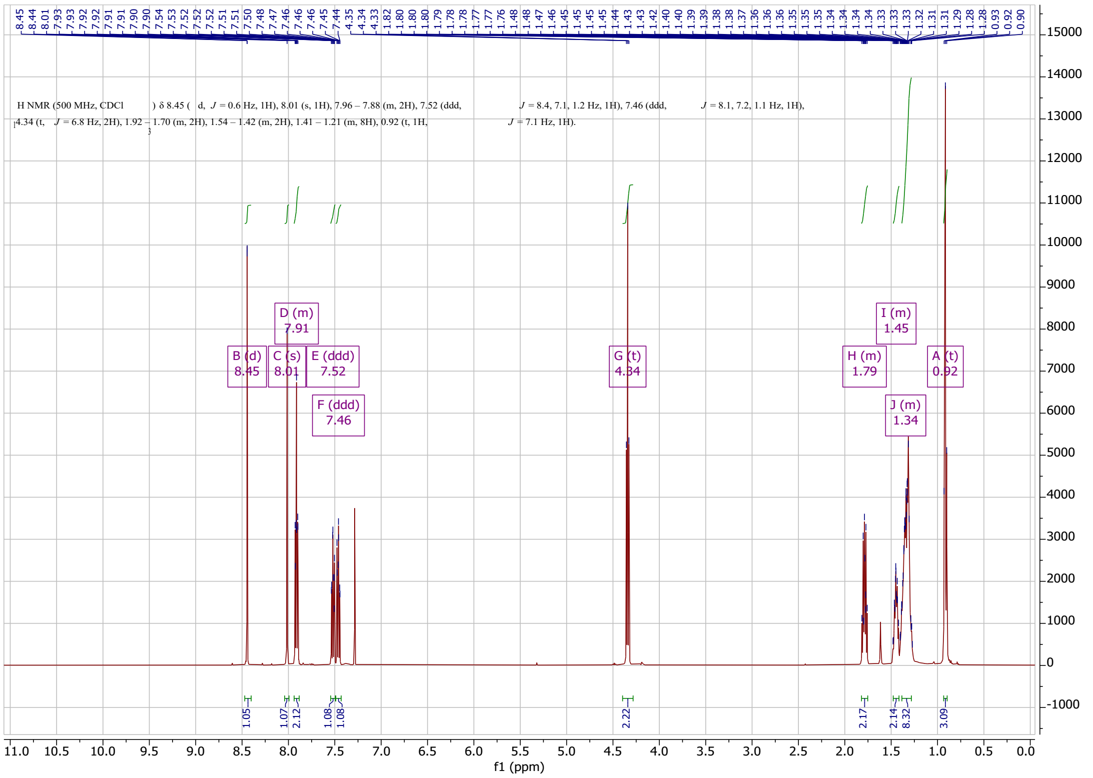

Spectrum S 5. ^{1}H NMR spectrum of (bztMe) (in CDCl$_3$).

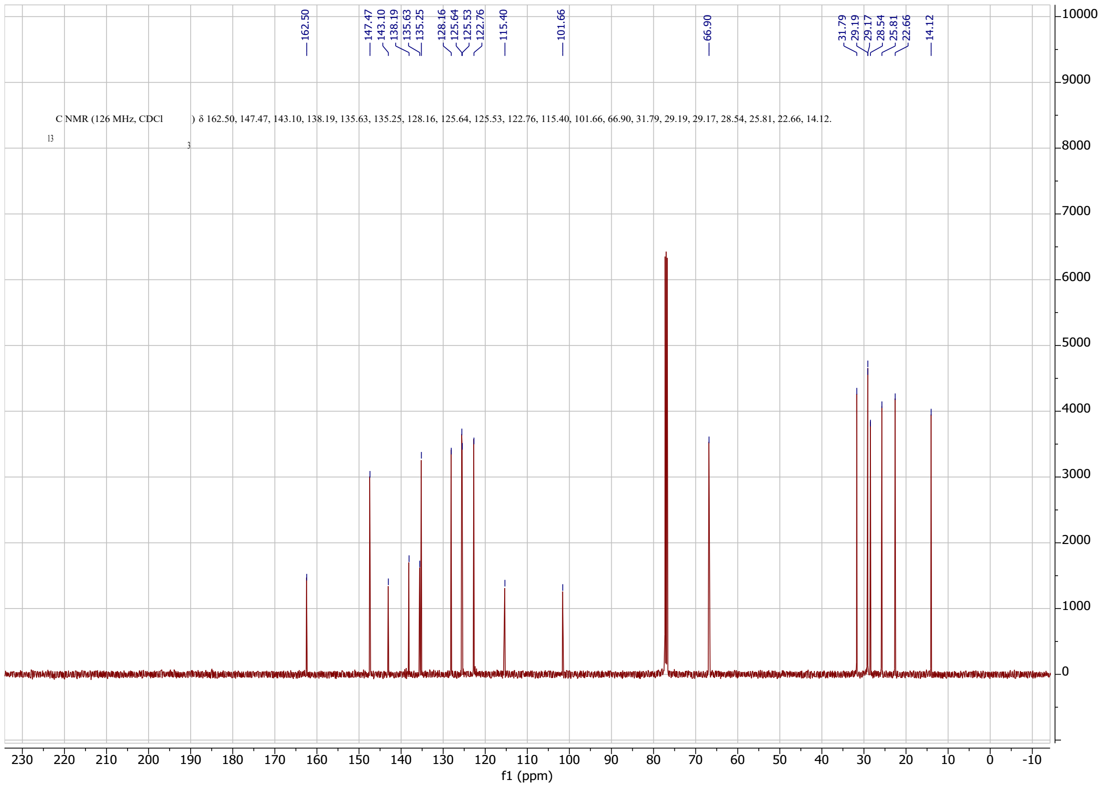

Spectrum S 6. ^{1}H NMR spectrum of (bztMe) (in CDCl$_3$).

2. Materials and Methods

2.1.Reagents and materials

General Methods: 1H and 13C NMR spectra were recorded on a Bruker Avance UltraShield spectrometer at 500 MHz. High-resolution mass spectrometry analyses were

performed on a Waters Xevo G2 Q-TOF mass spectrometer (Waters Corporation) equipped with an ESI source operating in positive-ion mode. UV/Vis spectra were recorded with a Biosens V5600 UV/Vis spectrophotometer. Emission spectra were recorded on an F-7000 HITACHI fluorescence spectrophotometer equipped with a Xenon lamp and Solid Sample Holder. Quantum yields Φ were obtained by the absolute method using an integrating sphere. The emission spectra were recorded by exciting the samples at their excitation spectral maxima unless otherwise mentioned. Electrochemical measurements were carried out with an Eco Chemie Autolab PGSTAT128n potentiostat using glassy carbon (with diam. 2 mm) as the working electrode, while a platinum coil and silver wire were used as the auxiliary and reference electrodes, respectively. The quantum theoretical calculations were performed with the use of density functional theory (DFT), with an exchange-correlation hybrid functional B3LYP and the basis set 6-311+G for all atoms. The calculations were carried out with the use of the Gaussian 16 program [1].

3. Optical properties

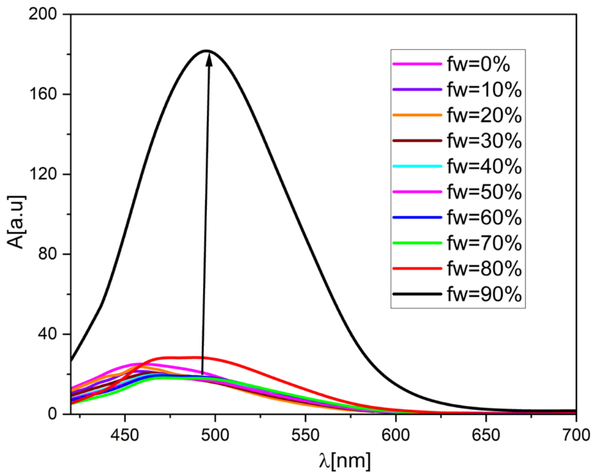

Figure S 1. PL spectra of the **bztMe** compound in MeCN/water solution (λ_{ex}= 360nm; c=1x10^{-5} M).

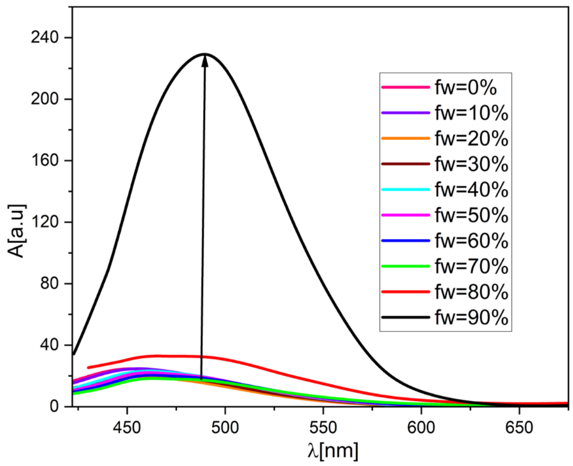

Figure S 2. PL spectra of the **bzttBu** compound in MeCN/water solution (λ_{ex}= 360nm; c=1x10^{-5} M).

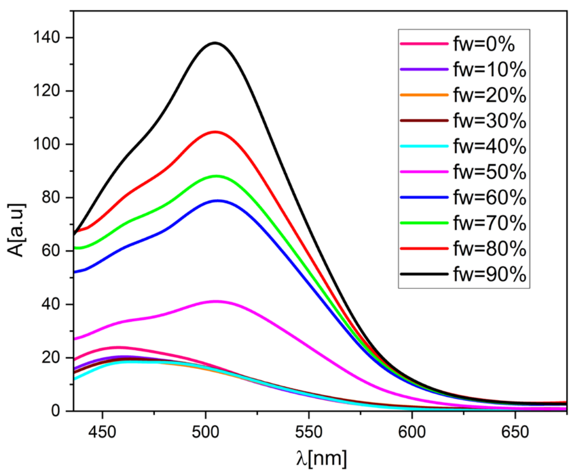

Figure S 3. PL spectra of the **bztOc** compound in MeCN/water solution (λ_{ex}= 360nm; c=1x10^{-5} M).

References

[1] Frisch, M.J.; Trucks, G.W.; Schlegel, H.B.; Scuseria, G.E.; Robb, M.A.; Cheeseman, J.R.; Scalmani, G.; Barone, V.; Petersson, G.A.; Nakatsuji, H.; et al. Gaussian 16, Revision C.01; Fox, Gaussian, Inc.: Wallingford, CT, USA, **2019**
